# Involvement of NtInvVR1 in the increase of reducing sugars during the curing of virginia tobacco

**DOI:** 10.1038/s41598-026-49747-x

**Published:** 2026-04-22

**Authors:** Aurore Hilfiker, Cecilia Cheval, Hong Ma, William A. Smith, Hélène Laparra, Joanne Schwaar, Delphine Langlet, Ralph E. Dewey, Simon Goepfert, Lucien Bovet

**Affiliations:** 1 PMI R&D, Philip Morris Products S.A, Quai Jeanrenaud 3, Neuchâtel, 2000 Switzerland; 2 Dept. of Crop and Soil Sciences, North Carolina State University, Raleigh, NC 27695-7620 USA

**Keywords:** Invertase, Tobacco, Sugars, Field experiment, Leaf curing, *NtInvVR1*, Biotechnology, Genetics, Molecular biology, Plant sciences

## Abstract

Flue-cured leaves of the Virginia tobacco (*Nicotiana tabacum* L.) market type are characterized by their high concentrations of sugars, namely sucrose (Suc), glucose (Glc), and fructose (Fru). During leaf curing, the so-called yellowing phase is associated with a dark-senescence process generating Suc and the main reducing sugars (RS), Glc and Fru. Invertase enzymes are reported to convert Suc into Glc and Fru throughout leaf senescence, but also fruit maturation. In this study, we actualize the complete family of genes encoding invertases (Invs) in *N. tabacum*. Two genes coding for vacuolar Invs, *NtInvVR1_S* and *NtInvVR1_T*, originating from the tobacco ancestors *N. sylvestris* and *N. tomentosiformis*, were identified to be the highest expressed *Inv* genes during the first 48 h of flue-curing (yellowing phase). Silencing both genes in the Virginia tobacco significantly reduced the accumulation of Glc and Fru by more than 90%, while Suc levels increased approximately 140% in a greenhouse environment. These data suggested that NtInvVR1_S and NtInvVR1_T play a key role in the hydrolysis of Suc, contributing to the accumulation of RS in cured leaves. NtInvVR1-RNAi plants were also cultivated in the field, showing a significant reduction in Glc and Fru of about 40% at the expense of Suc.

**Supplementary Information:**

The online version contains supplementary material available at 10.1038/s41598-026-49747-x.

## Introduction

Depending on the growing region and tobacco agricultural practices, once tobacco plants have reached maturity in the field, either single leaves or the entire stalk is traditionally harvested and subsequently transferred to a barn or an oven where the leaves are cured. Curing is a maturation process involving two successive steps: a yellowing phase and a drying phase. The specific curing processes performed post-harvest differ depending on the specific tobacco market type, with the Burley, Virginia, and Oriental market types being subjected to air-, flue-, and sun-curing, respectively. The yellowing phase starts shortly after harvest, and its duration varies according to the different curing processes. During this phase, the green leaves senesce, via a specific genetic program, giving a yellow coloration. In the subsequent drying phase, metabolic activities are greatly reduced due to the loss of water from the leaf tissue.

The flue-curing of Virginia tobaccos is performed by hanging or bulking leaves that have been stripped from the stalk in a heated, humidity-controlled barn for approximately one week. During leaf curing, a yellowing phase is followed by a drying phase, resulting in a significant alteration of the constituents via the induction of specific enzymatic activities^1–5^. During the yellowing phase, the genetic program shifts from being photosynthesis-driven to senescence-driven through the activation and inactivation of specific genes^6–8^. The leaf color change reflects processes associated with classical senescence, indicated by chlorophyll catabolism^9,10^ and the up-regulation of gene markers typically associated with senescence, such as senescence-activated genes (SAGs)^11^.

Among the main tobacco types, one distinguishing characteristic of Burley tobaccos is their requirement for high amounts of nitrogen (N)-fertilization (> 150 kg/ha) when grown commercially, which results in high levels of nitrate accumulation within the leaves^12^. In contrast, field-grown Virginia tobaccos require less N-fertilization (< 50 kg/ha). One indirect consequence of the differing nitrogen fertilization requirements between Burley and Virginia tobaccos is that flue-cured Virginia leaves typically retain high sugar levels after curing, often exceeding 10% of the total dry weight (DW)^6,13,14^. The sugars result from the hydrolysis of starch during the yellowing phase of curing^4,15^. However, the full pathway, including the specific genes and enzymes involved in converting starch to sugars, has yet to be elucidated. As a global apoptotic process, leaf senescence is a developmental stage aimed at sourcing reproductive organs with carbon sources, such as Suc^16,17^ and N-enriched compounds such as the aa, glutamine, and asparagine (Asn)^1,18,19^.

Sugars are naturally present in tobacco plants and are sometimes introduced as additives during the manufacturing of various tobacco- and nicotine-containing products. The sugars are mainly sucrose and inverted sugars and quantities may vary depending on the tobacco products. Sugars affect “desirable” product properties such as smoothness and flavor, mainly via Maillard reaction. On the other hand, sugars involve the formation of known toxicants and carcinogens as the result of thermal degradation, which occurs during product use^20–22^.

This study is aimed at understanding the molecular basis of sugar accumulation, and particularly RS production, in the leaves of flue-cured Virginia tobaccos^4,6^. The RS pool is primarily comprised of the hexose sugars Glc and Fru, which are formed from the hydrolysis of Suc, a reaction catalyzed by Inv enzymes^23,24^. In higher plants, the invertase (INV) gene family encodes enzymes that hydrolyze sucrose into glucose and fructose^25–27^, supplying carbon and energy while generating hexose signals that regulate growth and development^28^. Based on subcellular localization, INVs are classified into cell-wall (CWINs), vacuolar (VINs), and cytosolic invertases (CINs), each fulfilling distinct physiological roles. CWINs operate in the apoplast to drive phloem unloading^27,29^ and establish sink strength by converting incoming sucrose into readily imported hexoses^30,31^. VINs regulate vacuolar sugar storage^27,32,33^, osmotic balance^31^, and cell expansion^27^, thereby shaping the sucrose/hexose ratio in sink tissues^31,34^. CINs contribute to cytosolic sugar homeostasis^27^ and broader sugar-signaling functions^28,35^. Beyond carbon allocation, INV activity also impacts fruit flavor and quality by determining soluble sugar content and composition^36,37^. In fleshy fruits such as tomato, genetic modulation of CWIN pathways alters glucose and fructose accumulation^38^, increasing soluble solids and perceived sweetness^39^, perception^40^. Examination of the most up-to-date version of the allotetraploid tobacco genome and its progenitor species *N. sylvestris* and *N. tomentosiformis*^41^ revealed that the classification of tobacco genes coding for Invs, grouped into subfamilies encoding cell wall, alkaline/neutral, and vacuolar Inv enzymes^42^ was not accurate. We updated *Inv* gene categorizations by clustering *Inv* family members into a new phylogenetic tree grouped by homology with the Inv proteins identified in *S. lycopersicum* and *A. thaliana*. Of the 32 *Inv* genes found in the tobacco genome, we identified two closely related genes, *NtInvVRT1_S (N. sylvestris)* and *NtInvVRT1_T (N. tomentosiformis) copies*, that are highly expressed and actively induced during the yellowing phase of tobacco leaf curing. The silencing of both gene copies in tobacco affected the sugar content of cured leaves, as Glc and Fru levels were notably lower in cured leaves of plants grown in both greenhouse and field conditions. In both experiments, decreases in Glc and Fru were accompanied by a significant increase of Suc in *NtInvVR1*-RNAi plants compared to the control plants, strongly suggesting that *NtInvVR1_S/_T* gene products are directly involved in the hydrolysis of Suc during the early phase of the curing process.

## Results

### Generation of sugars during the early curing phase is associated with a senescence-driven process

**Fig. 1:**
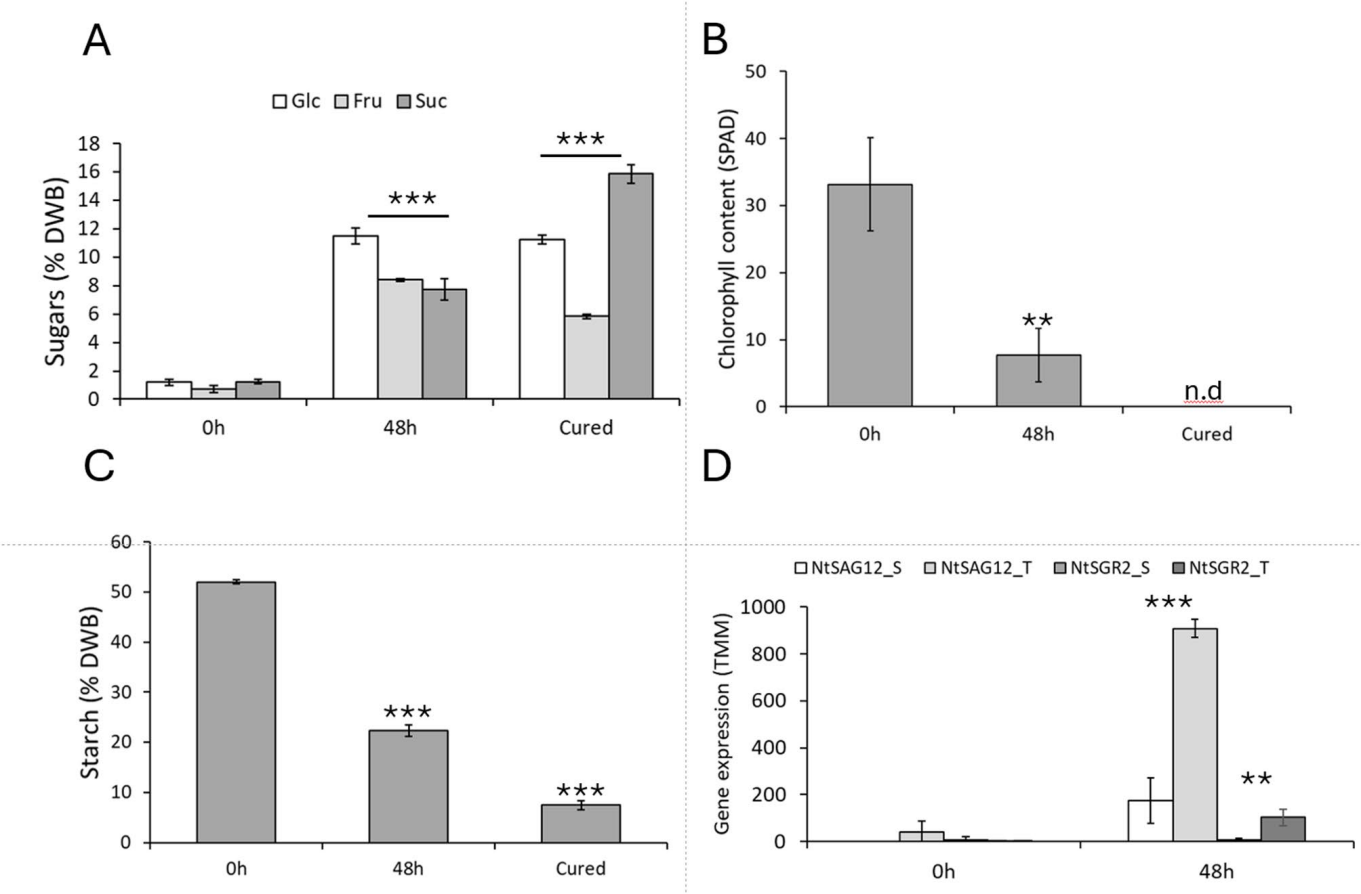
Changes in glucose (Glc), fructose (Fru), sucrose (Suc), chlorophyll, starch, *NtSAG12* and *NtSGR2* expression during leaf curing. For Glu, Fru, Suc (**A**), and starch measurements (**C**), leaves were collected in bulk replicates just before the initiation of curing (0 h), after 48 h of curing (48 h) and at the end of the curing (Cured). For sugars, starch and chlorophyll measurements, error bars represent biological standard deviation calculated from eight field-grown plants. Chlorophyll was determined in fresh leaves at the 0 h and 48 h time points using SPAD. (**B**). Samples collected at 0 h and 48 h were freeze-dried, and the mid-rib was removed before grinding and analysis. For *NtSAG12_S* (Ntab03g026240-1), *NtSAG12_T* (Ntab17g023410-1), *NtSGR2_S* (Ntab20g016560-1) and *NtSGR2_T* (Ntab15g016110-1) expression (**D**), RNAs were isolated from the leaf lamina at the 0 h and 48 h time points from three different plants. Statistical significance was assessed using Student’s t-test (**p < 0.01, ***p < 0.001). DW = dry weight; TMM = Trimmed Mean of M‑values (TMM) normalization method; SPAD = soil plant analysis development.

Virginia tobacco plants of the variety K326 were grown in a greenhouse environment where leaf samples were collected 58 days after transplanting (0 h, harvest time), after 48 h of curing (48 h), and at the end of curing (~ 170 h), when the leaves were fully dried. The main sugars, chlorophyll, starch content, and the expression of the senescence marker *NtSAG12 and NtSGR2* were determined in these samples. The data revealed that Glc, Fru, and Suc increased during the curing process, particularly during the first 48 h corresponding to the yellowing phase (Fig. 1A). The accumulation of sugars correlated with chlorophyll degradation (Fig. 1B). The sugar increase was associated with starch consumption (Fig. 1C) and a sharp rise in the expression of both S and T copies of *SAG12- and SGR2-*specific senescence markers (Fig. 1D). The observed changes in sugar and starch concentrations aligned with the results published by^4^. The degradation of chlorophyll can be attributed to the darkness-associated progression of leaf senescence^43^ that occurs during flue-curing^44^. *NtSAG12 and NtSGR2* are specific markers of leaf senescence^45–47^, previously shown to be activated during tobacco curing^6^.

### Phylogenetic analysis of NtInv gene family

**Fig. 2:**
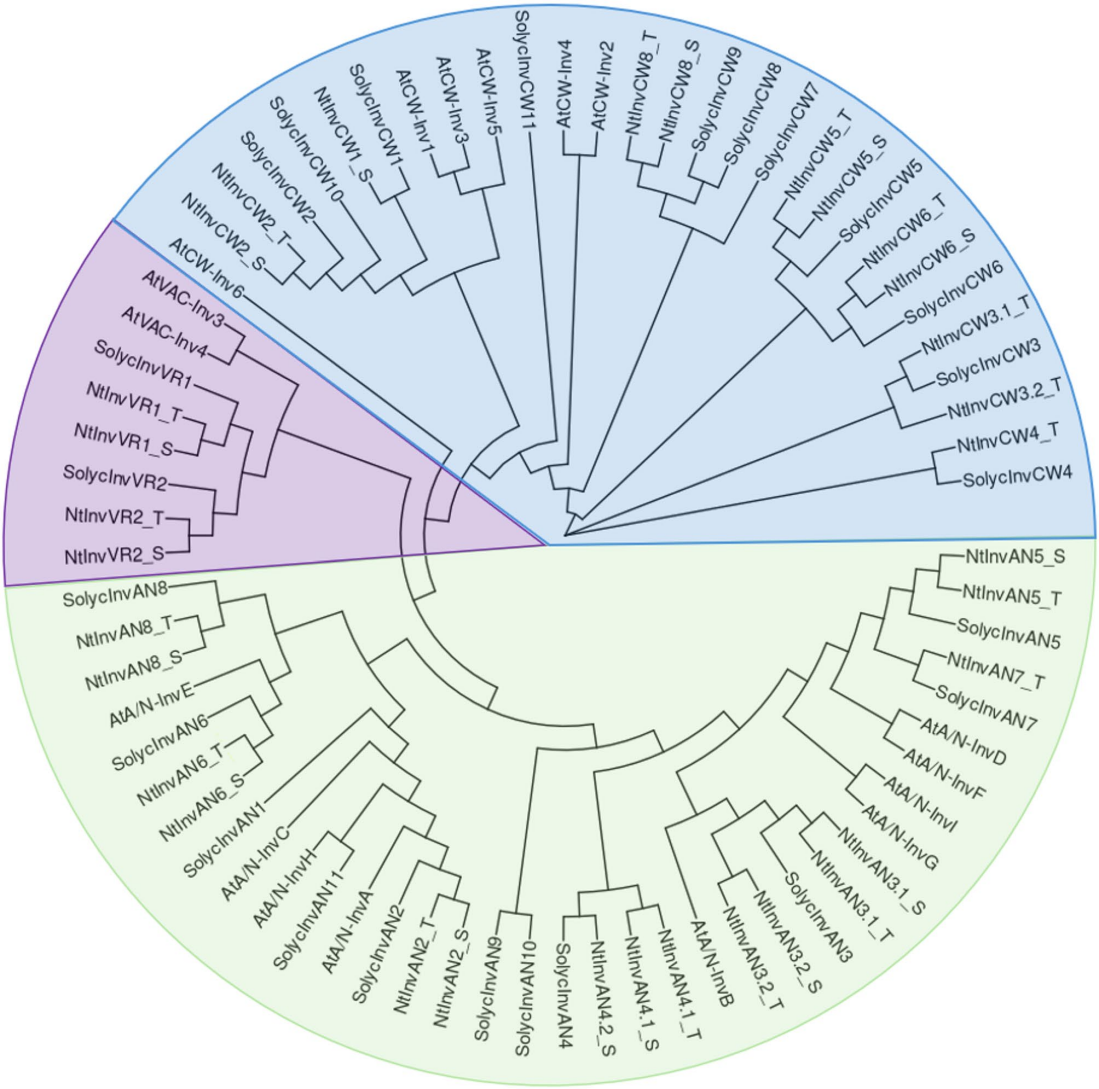
Phylogenetic tree of *Nicotiana tabacum* Inv protein family, including the corresponding Inv orthologs of *Solanum lycopersicum* and *Arabidopsis thaliana*. Nt: *Nicotiana tabacum*, Solyc: *Solanum lycopersicum*, At: *Arabidopsis thaliana*. Blue color highlights the cell wall invertase family, green color highlights the alkaline / neutral invertase family and the purple color highlights the vacuolar invertase family.

Phylogenetic analyses have revealed that *NtInv* gene products can be divided into 3 subgroups: alkaline/neutral, cell wall, and vacuolar Invs (Fig. 2).

Using the most recent genome assembly by Sierro et al.^41^, we identified 32 *NtInv* genes spread over 16 *N. tabacum* chromosomes (Supplementary Table S1). For the sake of uniformity, our tobacco gene nomenclature terminology was based on that used for the previously described tomato Inv family^48^. In comparison, there are 17 *Inv* genes in *A. thaliana* (Supplementary Table S2 in Tymowska-Lalanne and Kreis, 1998^49^) and 24 *Inv* genes in *S. lycopersicum* (Supplementary Table S3 in Fridman and Zamir, 2003^48^). As shown in Table 1, the largest subgroup in tobacco belonged to the alkaline/neutral category, consisting of 16 NtInv members split evenly between *N. sylvestris* (S) and *N. tomentosiformis* (T) as their ancestral origin. In this cluster, we can see one apparent duplication, NtInvAN4.1_S and NtInvAN4.2_S, and a single *N. tomentosiformis* derived copy for NtInvAN7_T. The second well-represented subgroup contained 12 members, belonging to the cell wall class of Invs. Among this subcluster, seven NtInvCW proteins originated from *N. tomentosiformis* and only five from *N. sylvestris*. One case, NtInvCW3.1_T and NtInvCW3.2_T, appears to be derived from gene duplication, while only a single copy of NtCW1, originating from *N. tometosiformis*, was found. The smallest subgroup was the vacuolar Invs, consisting of only four members, comprised of two genes, *NtInvVR1* and *NtInvVR2*, having one ortholog from *N. tomentosiformis* and the other from *N. sylvestris*. Interestingly, when considering that tobacco contains two genomes, the same core number of vacuolar Invs appears to be preserved among the three species *A. thaliana*, *S. lycopersicum*, and *N. tabacum*. Some studies suggest that vacuolar *Inv* genes, with their functions involved in cold tolerance, drought tolerance, and water stress, would be subject to a high degree of sequence conservation across species^42,50,51^. Although Cheng et al., 2023 reported 36 Inv gene products in the tobacco genome, we only identified 32 in our analysis^42^. The NtInvCW4-T and NtInvCW8-T proteins reported here were not identified by^42^, and six gene products (NtINV6, NtINV8, NtINV15, NtINV20, NtCWINV3, and NtCWINV6) reported in their study were not found in ours. This is likely the result of mis-assembly of the contigs prior to scaffolding. Figure 2 shows the rearranged phylogenetic tree of the *N. tabacum* Inv protein family, including the corresponding Inv orthologs of *S. lycopersicum* and *A. thaliana*.

The table presents gene names along with their expression levels measured in FPKM (Fragments Per Kilobase of transcript per Million mapped reads) for both 0 h and 48 h conditions. The log₂ fold change (log₂FC) represents the differential expression between the two time points (48 h vs. 0 h). Statistical significance is indicated by the q-value, which accounts for multiple testing correction using the false discovery rate (FDR) method.

**Table 1:** Gene expression analysis of *Nicotiana tabacum* Inv gene family between 0 h (0 h) and 48 h (48 h) conditions.

Invertase classes	Gene name	NCBI gene ID​	FC_T0h_lamina​	FC_T48h_lamina​	log_2_​	q-value​
Alkaline / Neutral​	NtInvAN2_S​	Ntab07g017700-1​	8.2​	18.67​	1.19​	0​
	NtInvAN2_T​	Ntab19g026160-1​	3.9​	22.49​	2.53​	0.03​
	NtInvAN3.1_S​	Ntab07g030630-1​	0.52​	0.18​	-1.48​	1​
	NtInvAN3.1_T​	Ntab19g016000-1​	1.35​	4.96​	1.88​	1​
	NtInvAN3.2_S​	Ntab06g004910-1​	0​	0​	0​	1​
	NtInvAN3.2_T​	Ntab04g025090-1​	11.49​	12.41​	0.11​	0.8​
	NtInvAN4.1_S​	Ntab01g007200-1​	0.08​	0​	-100​	1​
	NtInvAN4.1_T​	Ntab23g017730-1​	0.09​	0​	-100​	1​
	NtInvAN4.2_S​	Ntab12g001710-1​	20.07​	9.35​	-1.1​	0​
	NtInvAN5_S​	Ntab08g007440-1​	1.59​	1.85​	0.22​	1​
	NtInvAN5_T​	Ntab22g007280-1​	2.59​	1.18​	-1.13​	1​
	NtInvAN6_S​	Ntab11g004760-1​	6.4​	3.74​	-0.77​	1​
	NtInvAN6_T​	Ntab13g018800-1​	23.09​	19.59​	-0.24​	0.55​
	NtInvAN7_T​	Ntab04g029250-1​	0.57​	0.91​	0.67​	1​
	NtInvAN8_S​	Ntab18g015450-1​	7.9​	3.95​	-1​	0.04​
	NtInvAN8_T​	Ntab09g014720-1​	9.02​	14.66​	0.7​	0.06​
Cell Wall​	NtInvCW1​	Ntab06g025810-1​	0​	0.26​	100​	1​
	NtInvCW2_S​	Ntab08g000420-1​	0​	0​	0​	1​
	NtInvCW2_T​	Ntab22g007820-1​	0​	0.15​	100​	1​
	NtInvCW3.1_T​	Ntab10g000310-1​	0​	0.11​	100​	1​
	NtInvCW3.2_T​	Ntab10g000230-1​	0.43​	0.15​	-1.57​	1​
	NtInvCW4_T​	Ntab10g000300-1​	0​	0​	0​	1​
	NtInvCW5_S​	Ntab05g016060-1​	1.54​	0.75​	-1.03​	1​
	NtInvCW5_T​	Ntab24g016070-1​	18.85​	7.25​	-1.38​	0​
	NtInvCW6_S​	Ntab05g016080-1​	0​	0​	0​	1​
	NtInvCW6_T​	Ntab24g016090-1​	0​	0​	0​	1​
	NtInvCW8_S​	Ntab05g018980-1​	0.04​	0​	-100​	1​
	NtInvCW8_T​	Ntab24g015270-1​	0​	0​	0​	1​
Vacuolar​	NtInvVR1_S​	Ntab06g012900-1	6.8​	80.45​	3.57​	0​
	NtInvVR1_T	Ntab04g018310-1	20.63	127.73​	2.63​	0​
	NtInvVR2_S	Ntab01g004510-1	0.18	0​	-100​	1​
	NtInvVR2_T	Ntab23g020460-1	0	0​	0​	1​

The table presents gene names along with their expression levels measured in FPKM (Fragments Per Kilobase of transcript per Million mapped reads) for both 0 h and 48 h conditions. The log₂ fold change (log₂FC) represents the differential expression between the two time points (48 h vs. 0 h). Statistical significance is indicated by the q-value, which accounts for multiple testing correction using the false discovery rate (FDR) method.

### Protein sequence analysis of the NtInv family reveals a conserved domain among invertase proteins and a conserved N-terminus for vacuolar targeting

An analysis of the protein sequences of vacuolar Invs AtVAC-Inv3 and AtVAC-Inv4 in *A. thaliana* indicated a high degree of amino acid identity with other invertase proteins^52^. Comparative analyses of sugarcane cell wall and vacuolar (acid) invertases, including Inv Shcw1 (GenBank accession AY302084) and Inv ShinvA (GenBank accession AY302083), indicate strong homology across the protein length, with particularly high conservation within the GH32 invertase signature domain, reflecting their close evolutionary relationship^53^. Additional motifs are specifically characteristic of acidic Invs, including a conserved NDPNG(A) β-fructofuranosidase motif^54,55^, RDP and WECP(X)D motifs^56–59^, and the consensus sequence GVS[E/A]K^23,52,60^. Alignment of the four putative vacuolar invertases of *N. tabacum* (NtInvVR1/2_S/_T) with other vacuolar invertases confirmed the presence of these conserved motifs (Supplementary Table S4 and Fig. S1). The presence of these domains, especially the “WECXD” active site and “NPDNG” domain, is also conserved in yeast and bacteria^61^, strongly supports the classification of NtInvVR1/2_S/_T as acidic cellular invertases.

The vacuolar localization of NtInvVR1/2_S/_T enzymes is suggested by conservation of motifs found at the N-terminal region that have been associated with other demonstrated or putative vacuolar Invs from other species, including *A. thaliana* (At1g12240, At1g62660), *S. lycopersicum* (Solyc03g083910, Solyc08g079080)^27^, *Vitis vinifera* (Vitis02g00588, Vitis16g00881)^60^, *Zea mays* ZmINV1 (ZEAMMB73_Zm00001d002830)^56^ and sugarcane (*Saccharum hybrid* cultivar *Pindar*, SIA1)^53^. We performed a Signal P analysis (V2.0) but found no signal peptide. Further alignment of the N-terminal domain identified a putative transmembrane domain (TMD) using HMMTOP tools, suggesting membrane localization. A basic region adjacent to the membrane spanning domain and YXXØ motif was also found, a feature essential for vacuolar sorting^60,62^(Supplementary Fig. S1). These findings support the vacuolar localization and activity of NtInvVR1/2_S/_T in *N. tabacum*, consistent with observations in other species such as *Rosa hybrida*, where the membrane-anchored vacuolar invertase RhVI1 was shown to be highly expressed in petals and localized to the cytosolic side of the vacuolar membrane^63^.

### NtInvVR1_S and NtInvVR1_T expression are correlated Glc and Fru accumulation during leaf curing

We conducted an expression analysis of all *NtInv* genes during leaf curing, using the updated *NtInvs* gene list and new nomenclature (Table 1). Of the *Inv* genes showing baseline expression of > 1 FPKM at the initiation of curing, the most pronounced increases in expression after 48 h of curing were observed in the two *NtInvVR1* copies, *NtInvVR1_S* (11.8-fold increase) and *NtInvVR1_T* (6.2-fold increase). *NtInvAN2_S/_T* were also significantly induced after 48 h curing, but their overall expression levels were substantially lower than the two vacuolar *Invs* (18.67 and 22.49 FPKM, versus 80.45 and 127.73 FPKM). Due to their strong induction during curing, and high overall levels of expression, we postulated that the *NtInvVR1_S* and *_T* gene products would represent the best candidates for being directly involved in the cleavage of Suc and the production of Glc and Fru during leaf curing (Fig. 1). Interestingly, the second pair of vacuolar invertase genes, *NtInvVR2_S/* and _*T*, were not expressed during the curing process (Table 1), suggesting a clear differentiation in terms of function between the two vacuolar *Inv* gene pairs. Expression profiles of both *NtInvVR1_S/_T* and *NtInvVR2_S/_T* in different plant tissues supported this hypothesis. In addition to leaf tissue, *NtInvVR1_S/_T* were also expressed in roots, stems, immature flowers, sepals, and petals, with about two times expression observed in the non-photosynthetic petals (Supplementary Fig. S2). In contrast, minimal expression of *NtInvVR2_S/_T* was detected across all tissues assayed. To better assess the evolutionary relationships between tobacco *NtInvVR* genes and those from tomato and *A. thaliana*, we performed a phylogenetic comparison of *NtInvVR* encoded proteins across the three species. We found two protein sub-clusters that associated NtInvVR1_S/_T with SolycInvVR1 (Solyc03g083910), and NtInvVR2_S/_T with SolycInvVR2 (Solyc08g079080). The two *A. thaliana* proteins AtVAC-Inv3 and AtVAC-Inv4 (At1g12240, At1g62660) clustered more closely with each other than either of their tobacco or tomato VR1 counterparts (Supplementary Fig. S3).

**Fig. 3:**
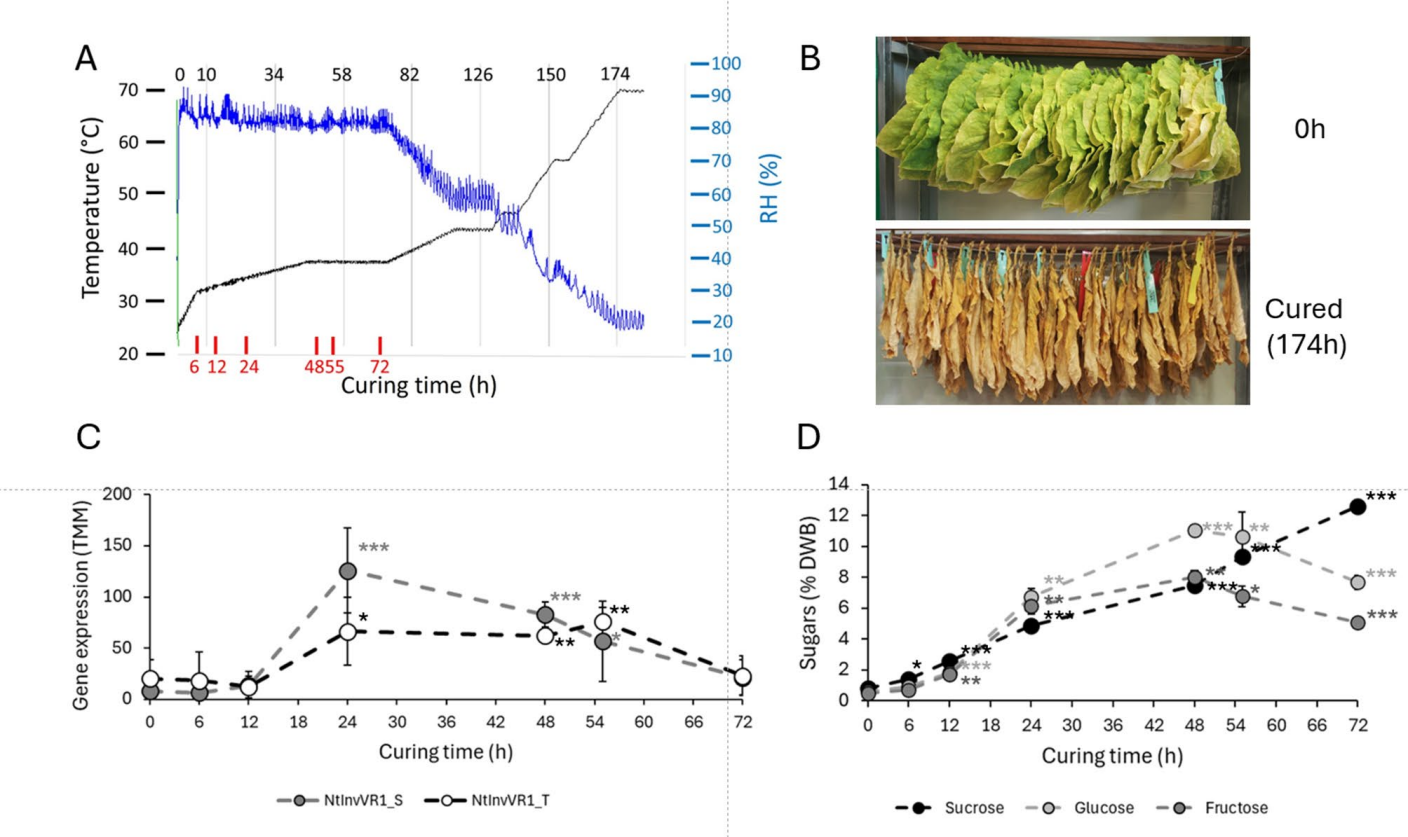
Expression levels of *NtInvVR1_S/_T* and sugar accumulation in the leaf lamina during the initial 72 h of curing. (**A**) Temperature (°C) and relative humidity (RH %, in blue) throughout the entire curing process (174 h). The times when tissue was collected for RNA-seq analysis are marked in red. (**B**) Image of the tobacco leaves before and after flue-curing; the upper picture was taken at 0 h (0 h) and the bottom picture shows the end of the flue curing cycle (cured, 174 h). (**C**) RNA-seq results (average of three leaf lamina samples, *n* = 3) showing the expression of *NtInvVR1_S/_T* (TMM) from 0 h to 72 h curing. The gene expression of *NtInvVR1_S* (gray dot) and *NtInvVR1_T* (white dot) at each time point was compared to T0 using T-test. (**D**) Measurements of sucrose (black dot), glucose (light gray dot), and fructose (dark gray dot) were performed in leaf lamina dried samples at the same time points as RNA-seq samples. Values expressed as % Dry Weight Basis (DWB) are presented as mean ± standard error (*n* = 3). Statistical analyses were conducted using a T-test, with *p < 0.05, **p < 0.01, ***p < 0.001.

A time-course experiment was performed to evaluate the expression of *NtInvVR1_S/_T* in comparison with sugar production during the first 72 h of curing. Figure 3A shows the changes in temperature and relative humidity (RH) within the flue-cured oven from the beginning of curing (0 h) to the end of the process (174 h). The relative humidity (RH) was relatively constant during the first 72 h, being maintained at around 82% during the yellowing phase. It subsequently decreased gradually to around 20% by the end of curing to facilitate the drying of the lamina and mid-rib. During the first 48 h, the temperature increased stepwise to reach 36 °C, where it was maintained through 72 h. Upon initiation of the drying phase, the temperature was again incrementally increased until reaching 70 °C by 174 h. Figure 3B shows the tobacco leaves before and after the curing was completed. Gene expression and sugar content in the leaf lamina were determined at different time points during the 72 h yellowing phase. *NtInvVR1_S* and *_T* expression progressively and significantly increased through 24 h of curing and then gradually decreased (Fig. 3C). A similar trend was observed for Glc and Fru, with a peak at 48 h, followed by a gradual decrease (Fig. 3D). In contrast, Suc continued to increase through 72 h. This suggests that the production of Suc, likely from starch (Fig. 1C), is active over a longer period than Glc and Fru originating from the Inv-mediated hydrolysis of Suc. The similarities between *NtInvVR1_S/_T* expression and Glc and Fru accumulation results support the proposal that NtInvVR1_S/_T functions to catalyze the hydrolysis of Suc during the curing of tobacco leaves.

To further address how sucrose derived from starch degradation may be redistributed during early curing, we examined the expression of selected sucrose and hexose transporter genes belonging to SUT(SUC) and SWEET family members in *Nicotiana tabacum*. The sequences were identified based on protein sequence homology to Arabidopsis SUT^64^ and SWEET family members^65–67^. *NtSUT2* and *NtSUT5* transcripts showed down-regulation during curing, while *NtSUT7* transcripts which the orthologues of *AtSUT4* increased later to 48 h (Supplementary Table S5). This is not surprising since *AtSUT4* is reported to be induced by sucrose^68^. In parallel, multiple SWEET family members, including orthologues of *AtSWEET11*, *AtSWEET12*, and *AtSWEET13* were also strongly upregulated after 24 h curing in *Nicotiana tabacum* (Supplementary Table S6).

### Impact of RNAi-mediated suppression of NtInvVR1 on sugar accumulation in cured leaves of greenhouse and field-cultivated plants

**Fig. 4:**
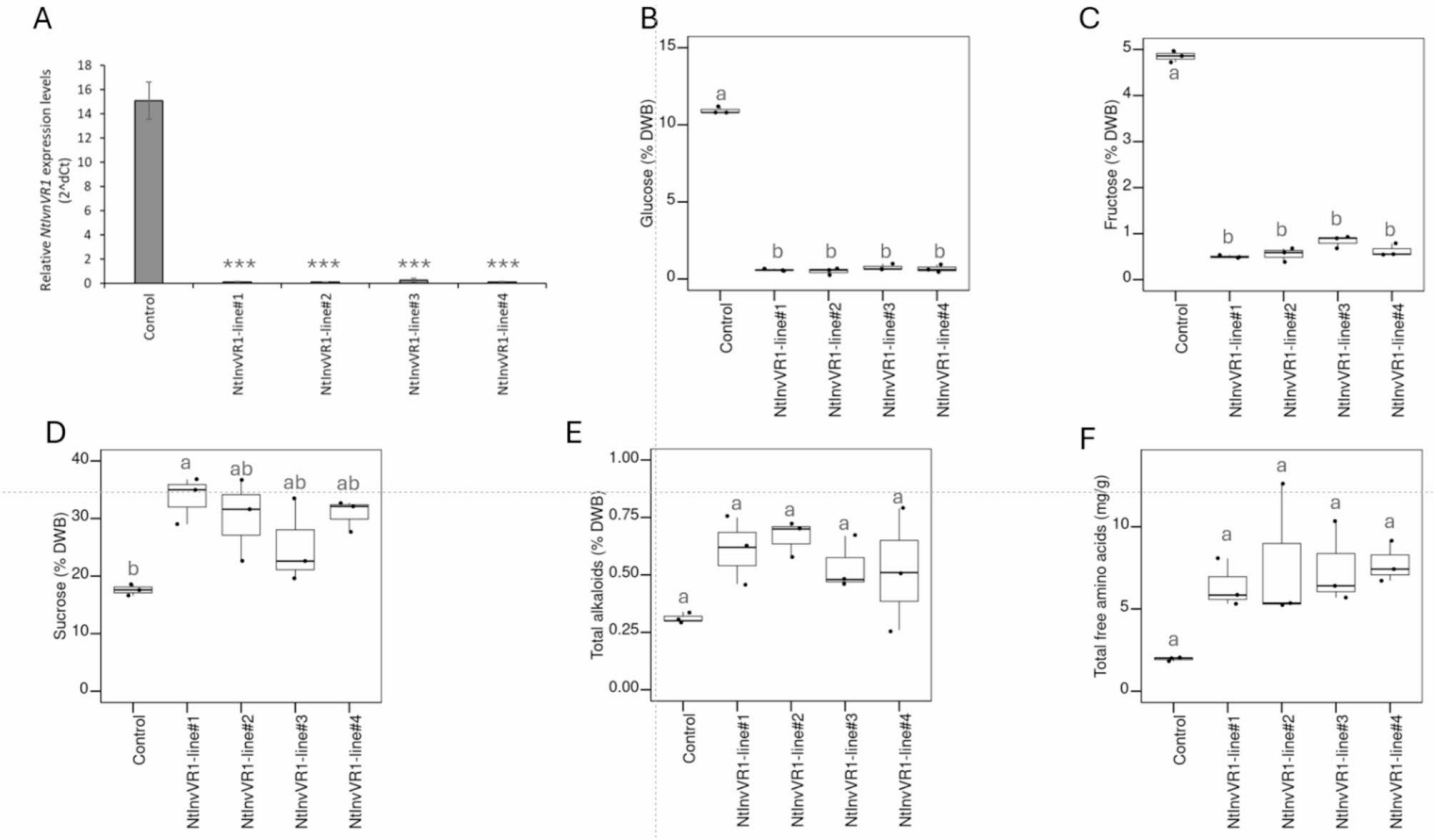
Analysis of greenhouse grown NtInvVR1-RNAi plants. *NtInvVR1_S/_T* transcript levels after 48 h of leaf curing (**A**) and the analysis of glucose (**B**), fructose (**C**), sucrose (**D**), total alkaloids (**E**), total free amino acids (**F**) content after flue curing in control and NtInvVR1-RNAi plants. Three representative T1 plants (*n* = 3) of four independent NtInvVR1-RNAi lines and controls were grown using a randomized design. Values represent the mean ± standard error of tree biological replicates. For *NtInvVR1* expression levels (**A**), dCt = delta Ct analysis, statistical analyses were performed using Student’s t-Test (unequal variance), ***p < 0.001, bars represent the standard error of three biological replicates. For metabolomic, statistical analyses were performed using the Krustal Wallis and posthoc test, *p* < 0.05, bars represent the standard error of three biological replicates.

We used a genetic approach to determine the impact of *NtInvVR1_S/_T* on sugar levels in cured leaves of plants of Virginia tobacco variety K326. Four independent NtInvVR1-RNAi lines and controls were grown in a controlled greenhouse environment. At maturity (58 days after transplanting), leaves were subjected to curing, and after 48 h, qRT-PCR was conducted to measure *NtInvVR1_S/_T* transcript levels. As shown in Fig. 4A, *NtInvVR1_S* and *NtInvVR1_T* transcripts decreased by as much as 95% in each independent NtInvVR1-RNAi line relative to controls. In the same plants, the content of Glc and Fru levels decreased dramatically and significantly by 94% and 87%, respectively (Fig. 4B,C), whereas the Suc content significantly increased, almost doubling in some RNAi lines in comparison to controls (Fig. 4D). Although the mean alkaloid and amino acid contents of the *NtInvVR1*-RNAi lines were greater than that observed in the control line, these differences were not considered to be statistically significant (Fig. 4E and F). In the control plants, the cured lamina contained approximately 50% Suc, and 50% Glc + Fru under greenhouse growth conditions. In the NtInvVR1-RNAi plant lines, however, the total sugars in the cured lamina contained more than 94% Suc and less than 6% of Glc + Fru, thereby indicating that the silencing of *NtInvVR1_S/_T* hindered the accumulation of RS through the inability to metabolize Suc (Supplementary Table S7).

**Fig. 5:**
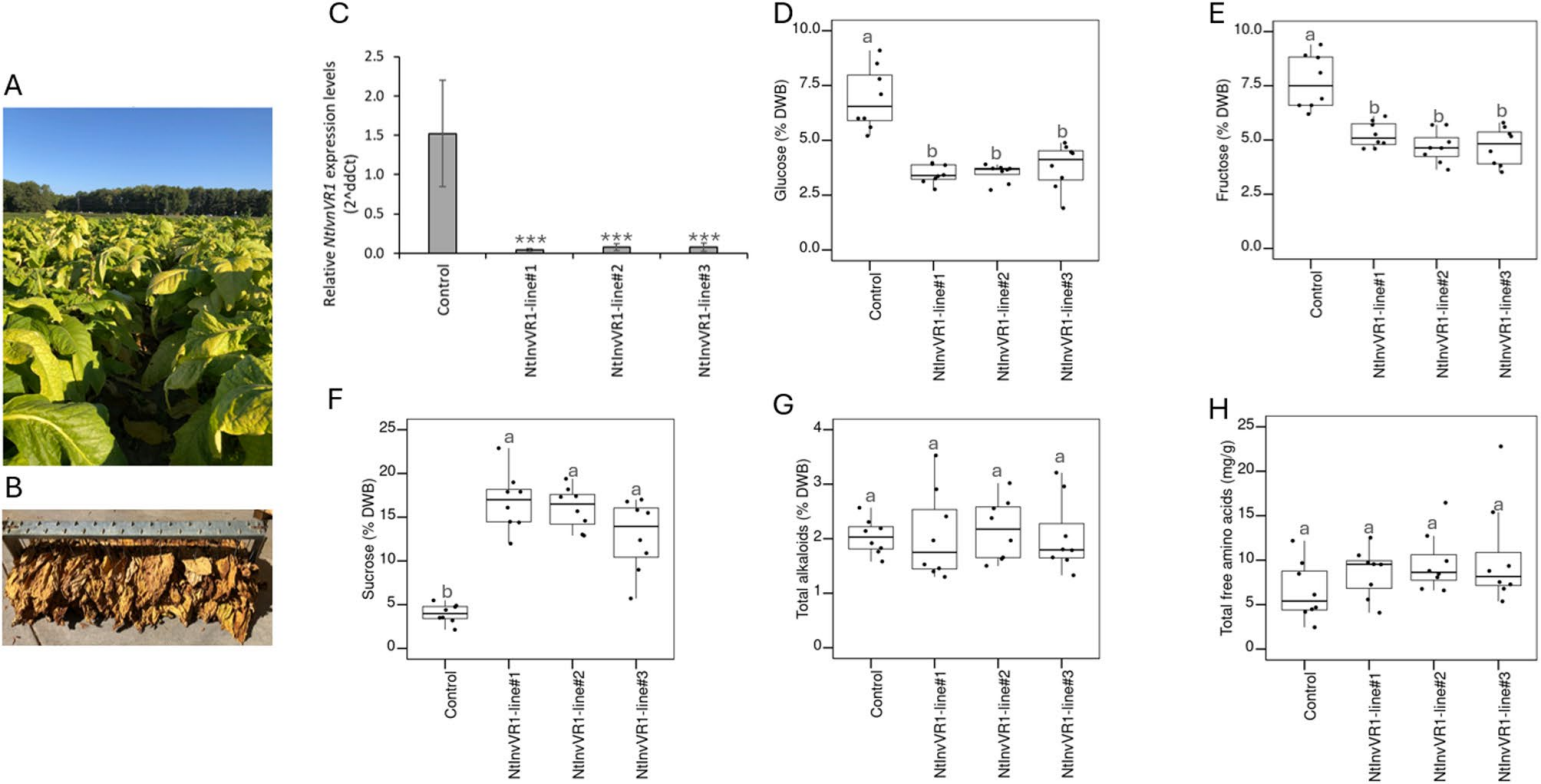
Field grown phenotypes of NtInvVR1-RNAi plants. Plants (random repartition of controls and NtInvVR1-RNAi) before harvest (**A**) and bulked leaf curing (**B**). *NtInvVR1_S/_T* transcript levels after 48 h of leaf curing, ddCt = double delta Ct analysis, control, line#1 and #2 *n* = 12, line #3 *n* = 8. Statistical analysis was performed using Student’s t-Test (unequal variance), *** *p* < 0.001. Bars represent the standard deviation of biological replicates. (**C**), glucose (**D**), fructose (**E**), sucrose (**F**) total alkaloids (**G**) and total free amino acids (H) contents after full curing in wild-type (control) and NtInvVR1-RNAi plant lines. Values (*n* = 8) represent the mean ± standard error of the biological replicates; statistical analyses were performed using the Krustal Wallis and posthoc test, *p* < 0.05.

NtInvVR1-RNAi lines were planted in a field in the US (Clayton, NC) (Fig. 5A), using a randomized complete block design, to establish the degree to which suppression of *NtInvVR1_S/_T* impacted the sugar content in cured leaves. At maturity, two leaves from each plant were placed in a flue-curing structure for 8 days. This flue cured material (Fig. 5B) was then used for sugar and other metabolites quantification. Similar to the greenhouse grown plant, the *NtInvVR1* transcripts were also strongly and significantly reduced in the three *NtInvVR1*-RNAi lines planted compared to controls (Fig. 5C). In the same lines, the Glc (Fig. 5D) and Fru (Fig. 5E) levels were also significantly reduced in cured leaf lamina of NtInvVR1-RNAi plants compared to controls, as was observed in the greenhouse. Suc levels were also significantly higher in the transgenic lines compared to control plants (Fig. 5F) without any impact on total alkaloids (Fig. 5G) and total aa (Fig. 5H).

In the cured lamina of the control plants, sugars were partitioned as approximately 20% Suc and 80% of Glc + Fru. In contrast, in the NtInvVR1-RNAi plant lines, sugar accumulation in the cured lamina was comprised of more than 60% Suc and less than 40% of Glc + Fru, thereby indicating that the silencing of *NtInvVR1_S/_T* reduced the ability of the leaf to convert Suc to RS (Supplementary Table S8).

Under both greenhouse and field conditions, NtInvVR1 RNAi plants did not exhibit obvious differences in overall growth or development compared to control plants based on visual and fresh weight comparisons.

## Discussion

Based on their subcellular localization, invertases (INVs) can be classified as acidic or neutral/alkaline enzymes. Their expression is organ-specific and/or developmentally regulated, enabling them to fulfill distinct functions at different stages of plant growth and development. INV activity is tightly regulated by plant hormones and environmental cues, thereby modulating sucrose transport and phloem unloading through sucrose hydrolysis. By reducing sucrose concentrations in the apoplast, invertases maintain the concentration gradient required for efficient sucrose unloading. Consequently, changes in the reducing sugar (RS) pool may alter osmotic balance and water flux within plant organs^25,28,32^, ultimately influencing dehydration dynamics during flue-curing.

The origin of Suc, Glc, and Fru accumulation in flue-cured Virginia tobaccos has not been fully resolved. It is generally accepted that these sugars are products of starch degradation. Although the details of the specific starch degradation pathway that is activated during leaf curing have not been identified, considering that the yellowing phase shares hallmarks of senescence, starch is likely to be catabolized via a complex process, producing Suc as the final product^69^. Depending on the plant species and growth conditions, a large proportion of the fixed carbon is stored as starch within the chloroplasts^70^. Suc is a degradation product of starch^71^, but the pathway leading to Suc production from the catabolism of starch is complex and highly regulated, involving more than 15 different enzymes^69,72^. Senescing leaves are considered source organs, delivering Suc into the phloem for transport to sink organs, such as flowers, seeds, and tubers^73^. When Suc is produced in the cytoplasm from starch degradation, it can be either transiently stored and accumulated to high levels within the vacuole or transported to the apoplast in a pre-phloem loading step^74–76^. Suc is a disaccharide that can be hydrolyzed into Glc and Fru by specific Inv enzymes found in three distinct sub-cellular localizations: the cell wall (CW-Inv), the cytoplasm (C-Inv), or the vacuole (V-Inv)^77^.The fate of Suc as a carbon source during natural leaf senescence is the export to the sink organs via the apoplast^16,74^ or the import into the vacuole through the tonoplast, where V-Inv activities can catalyze its hydrolysis. In addition to the increased expression of *NtInvVR1_S/_T* during the early curing phase, the expression of several transporters orthologous to *AtSUT4* and *AtSWEET11–13* also rose between 24 and 48 hours^69^. This result suggests that a coordinated transcriptional regulation of these genes occurs during this senescence process, which may lead to a cellular relocalization of sucrose and hexoses.

The accumulation of Suc and RS that occurs during tobacco leaf curing may share similarities with observations made during fruit maturation in certain *Solanacea* species. In sugar-storing sink organs such as fruit, high acid Inv activity appears to be correlated with the accumulation of hexoses. In the Suc-storing fruit of the wild tomato species *Lycopersicon chmielewskii*, Inv activity is greatly reduced, whereas in the hexose-storing fruit of domestic tomato (*S. lycopersicum*), Inv activity increases at the onset of fruit ripening^78^. The reduction of Inv activities by antisense^34^ or co-suppression^79^ led to the conversion of hexose-storing fruits into Suc-storing fruits, demonstrating that in tomato such an Inv plays a pivotal role in controlling sugar composition. Such an observation seems to be similarly applicable to tobacco leaf yellowing during curing.

The physiological shift that occurs during tobacco leaf curing is associated with a rapid loss of chlorophyll pigments as the leaves transition to non-photosynthetic organs, like fruit or petals. Interestingly, the expression of *NtInvVR1_S/_T* and four *ASN* genes^1^ is not only strongly induced during the senescence phase of curing in leaves, but are also highly expressed in non-photosynthetic petals. Their respective gene products catalyze the formation of RS and Asn, as carbon and N resources for sink organs^80^.

Dehydration is also a hallmark of the flue-curing process, as the moisture content in tobacco leaves drops from approximately 75% to less than 15%^15^ and the induction of genes associated with drought stress responses is observed^6^. Immediately after harvest, the leaf begins to wilt and starts yellowing within a couple of hours, thereby stimulating the production of osmoprotectants, including sugars^81,82^ and some amino acids like proline^83–85^. Osmoregulation is key in maintaining cellular activities during dehydration. The cleavage of Suc into Glc and Fru may greatly increase the osmotic pressure of cells^26^. Altogether, our observations suggest that the production of Glc and Fru takes place during the first 72 h of leaf curing via the activity of NtInvVR1_S/_T enzymes and thus may play a role in the osmoregulation process, in addition to proline and other dehydration-associated proteins such as dehydrin and LEA4, as observed in tea plants (*Camellia sinensis L.*)^50^.

The results of this study also suggest that the impact of *NtInvVR1_S/_T* suppression appeared to differ when the plants were grown under the controlled conditions of a greenhouse, compared to a field environment. In the greenhouse, the production of Glc and Fru was drastically reduced in the NtInvVR1-RNAi plants, suggesting that NtInvVR1_S/_T are part of key enzymes involved in the hydrolysis of Suc when the leaves are being cured. In addition, the Suc content significantly increased in these same plants, providing strong evidence that Glc and Fru result from the direct hydrolysis of Suc catalyzed by NtInvVR1_S/_T. However, under field conditions, the impact of *NtInvVR1_S/_T* silencing was less marked, though still significant. For example, we found in our control plants that Suc accumulation (%DW) was four times higher under greenhouse conditions compared to the field. It is possible that Suc is channeled differently under field conditions, where more Suc may be transported to non-vacuolar cytosolic or apoplastic compartments (source/sink), and thus not accessible to NtInvVR1_S/_T but to other Inv enzymes^27^, such as NtInvAN2_S/_T which were also somewhat upregulated during curing. Differences between the levels of starch at leaf maturity, when curing is initiated, may also account for differences between greenhouse and field data. The variations observed between greenhouse and field results may also originate from the curing process. In the greenhouse, curing conditions are highly controlled, including a consistent flue-cured barn filling rate and stable external temperature. These parameters are not maintained in the field. Consequently, the rate of yellowing and drying during the first 72 h of curing can be affected, which in turn may influence the activities of NtInvVR1_S/_T.

The quality of Virginia tobacco leaves is highly dependent on the leaf chemistry changes that occur during the curing process, with the accumulation of RS being among the most notable. Differences in NtInvR1_S/_T activities may change the quality of the commercial material by altering the taste and sensory properties of the final products. This is especially relevant given that Virginia tobaccos represent the largest proportion of the tobacco market.

## Conclusion

Strong genetic and physiological shifts occur after mature tobacco leaves are subject to curing. Green leaves undergo senescence-like changes, which are accelerated and controlled by the farmers in specialized barns to ensure the quality of cured leaf. Quality is directly related to the leaf chemistry profile, which in turn, is dependent upon changes in the array of enzymes present in the leaf during the curing process. Flue-cured Virginia tobacco leaves are characterized by a high sugar content, which may reach up to 20% DW. As a product of the catabolism of amylose and amylopectin, Suc is a pivotal carbon compound in plants. Its accumulation and degradation to Glc and Fru during the yellowing phase of curing is of key importance in sustaining tobacco leaf quality. This study shows that the activities of NtInvVR1_S/_T play a noticeable role in contributing to the unique chemistry profile of cured Virginia tobaccos with respect to the accumulation of sugars. Orthologs of *NtInvVR1_S/_T* are likely active in the leaves of other commercial commodities, such as aromatic plants, and thus may contribute toward the distinguishing chemistry profiles that define their unique properties as well. Finally, it is possible that an increase in Suc in naturally senescing leaves, driven via the inactivation of V-Inv orthologs, may contribute to enhancing the source-sink flow of this important carbon compound in plants, potentially impacting seed, fruit, and flower quality.

## Methods

### Plasmid constructs

The specific DNA exon fragment selected for suppressing the expression of both copies of *NtInvVR1* (*NtInvVR1_S* and *NtInvVR1*_T) was:

5′-GGTTTCAACTGCAGTACTAGTGGAGG

TGCTGCTAAAAGAGGCATTTTGGGACCATTT

GGTGTCGTTGTAATTGCTGAT CAAACGCTTTCTGAGCTAAC-3′. This DNA fragment of 98-bp exhibiting 100% identity to both *NtInvVR1* sequences was cloned in both sense and antisense orientations (separated by an intron) between the strong constitutive Mirabilis Mosaic Virus (MMV) promoter and the 3′ nos terminator sequence of the nopaline synthase gene of *Agrobacterium tumefaciens*^86^ within a PMI-proprietary binary vector (WO2012098111). The RNAi cassette should concomitantly suppress the expression of both *NtInvVR1_S* and *NtInvVR1_T* and was introduced into K326 using Agrobacterium-mediated transformation for constitutive expression.

### Plant materials, growing conditions, and leaf curing

For the curing time-course analyses, seeds of tobacco variety K326 (PI 552505, TC 319, USDA-GRIN database) were sown on soil-containing float trays (float tray solution: Hauert Plantaaktiv 15 + 7 + 22, Hauert, Grossaffoltern, Switzerland). Two weeks after germination, seedlings were individualized and grown for an additional 4 weeks under a 16 h (24 °C):8 h (18 °C) light: dark cycle regime. Plantlets were transferred into 5 L pots and fertilized daily with media purchased from Yara Benelux B.V. (Vlaardingen, the Netherlands) containing 666.5 mg NO_3_, 18 mg NH_4_ (total of 165.39 mg N), 88.78 mg P_2_O_5_, 306.25 mg K_2_O, 49.99 mg Mg, 185.61 mg Ca, 369.60 mg SO_4_, 0.839 mg Fe, 0.549 mg Mn, 0.262 mg Zn, 0.216 mg B, 0.048 mg Cu, and 0.048 mg Mo per liter. All plants were grown using the same 16 h (24 °C):8 h (18 °C) light: dark cycle.

For the *NtInvVR1* suppression studies, leaf discs from young K326 plants were transformed using a standard *Agrobacterium*-mediated transformation protocol^87^. Once the T_0_ plants were transferred into the greenhouse, they were maintained using the same fertilization and culture conditions described above. The leaf curing was performed in a curing oven (Ventobacco curing barn, Vencon Varsos S.A., Athens, Greece) as described in^88^. Forty-eight hours after the initiation of curing, leaves from 20 independent T_0_ plants were assayed by qRT-PCR to select those with the most greatly RNAi-mediated inhibition of the targeted genes. Seeds were harvested from four independent T_0_ lines that exhibited the strongest *NtInvVR1* silencing.

T_1_ generation seeds of *NtInvVR1*-RNAi plants and their respective controls were surface sterilized with chlorine gas and sown under axenic conditions on Murashige and Skoog (MS) medium including vitamins and MES-buffer (Duchefa Biochemie B.V., Haarlem, The Netherlands), supplemented with 2% Suc (Duchefa Biochemie B.V.), and 0.8% Phyto Agar (Duchefa Biochemie B.V.). The agar plates also contained 100 mg/L^− 1^ kanamycin (Duchefa Biochemie B.V.) to eliminate non-transgenic segregants. Plants were transferred from selection media to float trays in the greenhouse, where they were grown for an additional 6 weeks before transplantation into 5 L pots (see fertilization and culture conditions above). After a 48-hour curing period, leaves from T_1_ plants and their respective controls were assayed for sugar content, as well as by qRT-PCR to assess *NtInvVR1* suppression via RNAi.

### Phylogenetic tree

Phylogenetic analysis was conducted using R (version 4.2.3) and several specialized packages for sequence alignment, distance matrix computation, and tree construction. The following R packages were employed: ggplot2 (version 4.2.3) for data visualization, ape (version 5.7.1) for phylogenetic analysis, ggtree (version 3.6.2) for tree visualization, seqinr (version 4.2.30) for reading and manipulating sequence data, phangorn (version 2.11.1) for phylogenetic reconstruction. Protein sequences were aligned using the Clustal Omega algorithm (version 1.2.4) via the EMBL-EBI web server, using the --full --full-iter options to enable full distance matrix computation and iterative refinement for improved alignment accuracy, and the resulting alignment was saved in FASTA format. The alignment was read into R using the read.alignment() function from the seqinr package. A pairwise similarity matrix was computed using dist.alignment() and converted into a distance object. A phylogenetic tree was then constructed using the Neighbor-Joining with Subtree Pruning and Regrafting (NJS) method via the njs() function from the ape package. The sequences utilized are available at: https://www.solgenomics.net/organism/Nicotiana_tabacum/genome^41^ for *Nicotiana tabacum*, and National Center for Biotechnology Information (NCBI) at: https://www.ncbi.nlm.nih.gov/ for *S. lycopersicum* and *A. thaliana* protein sequences.

A multiple sequence alignment of *Inv* vacuolar orthologs was generated using Clustal Omega (version 1.2.4, with the options --full–full-iter parameters), and a phylogenetic tree was constructed from these alignments by using FastTree (version 2.1.10), with the options-gamma-pseudo-spr4-mlacc2-slownni). The resulting tree was re-rooted on the longest branch and ordered on the leaf labels while preserving the tree topology by using the nw_re-root and nw_order programs from newick_utils (version 1.6) and visualized using ete3 (version 3.1.1). Labels on the nodes are local support values calculated using the Shimodaira–Hasegawa test. The sequences utilized are available at: https://www.solgenomics.net/organism/Nicotiana_tabacum/genome^41^.

### Protein sequence alignment

A multiple protein sequence alignment of Inv vacuolar orthologues was generated using the CLC Main Workbench alignment and tree tools from Qiagen^®^ (www.qiagen.com). Protein sequence for *A. thaliana* (At1g12240, At1g62660), *S. lycopersicum* (Solyc03g083910, Solyc08g079080), *Vitis vinifera* (Vitis02g00588, Vitis16g00881), *Zea mays* ZmINV1 (ZEAMMB73_Zm00001d002830) and sugarcane (*Saccharum hybrid* cultivar *Pindar*, SIA1) are available at: https://www.ncbi.nlm.nih.gov/protein. *Nicotiana tabacum* protein sequences utilized are available at: https://www.solgenomics.net/organism/Nicotiana_tabacum/genome^41^.

### RNA-seq analyses with field-grown K326

Virginia variety K326 plants were grown in a Swiss field (silt-clay soils, pH 7.8). After flowering (60 days after transplanting), petals, sepals, immature flowers, stems, roots, and upper, middle, and lower leaves were collected in triplicate and flash-frozen in liquid N_2_. The data represent the averages from RNA isolated from three independent plants (*n* = 3) grown in a field in Switzerland as described in Bovet et al., (2022)^89^. Lamina samples were collected during leaf curing at start (0 h) and after 6 h, 12 h, 24 h, 48 h, 55 h, and 72 h treatment. Total RNA was isolated from each tissue sample (*n* = 3) using the RNeasy mini kit (Qiagen, Hilden, Germany). Prior to cDNA library construction, RNA purity and integrity were checked using a NanoDrop spectrophotometer (Qiagen). The mRNA was enriched from total RNA by using a polyA column to generate cDNA libraries for sequence analysis. All libraries were sequenced on an Illumina HiSeq-2500 sequencer (Illumina, San Diego, CA, USA) using version 3 chemistry and flow cells with runs of 2 × 100 bases. The reads were cleaned using Trimmomatic (version 0.32^90^) and aligned to the tobacco genome using Hisat2 (version 2.0.1 beta^91^). Cuffdiff2 (version 2.2.1^92^) was used to calculate the FPKM (fragments per kilobase of transcript per million fragments mapped) values based on the aligned reads. In parallel, RNA-seq data were also normalized using the trimmed mean of M-values (TMM) method to allow complementary expression analyses. The transcriptomes were derived by mapping RNA-Seq reads from each tissue to the tobacco reference genome (https://www.solgenomics.net/organism/Nicotiana_tabacum/genome). Three biological replicates were obtained for each plant and tissue. Previously published gene models were used as the basis for differential gene expression analysis^93^.

### qRT-PCR analysis

The effectiveness of the RNAi constructs in silencing *NtInvVR1_S/_T* expression was assessed by qRT-PCR. RNA was isolated from each independent T_0_ transformation event and their corresponding control plants; qRT-PCR was performed using primers (5′- to 3′-) *NtInvVR1*-F: caaacgggttggacacatcatataac, *NtInvVR1*-R: ggttatcaggagtccatttgttcttt for the targeted genes and *NtGS2*-F: ggaggctaccccggacct, *NtGS2*-R: caagccttgtagtgagcatct, for the reference gene. Each PCR reaction contained 0.01 µM of forward and reverse primers, 2 µL of cDNA, and 5 µL of PowerUp™ SYBR™ Green Master Mix ThermoFisher Scientific) in a 10-µL reaction mix. The reaction mix was heated to 95 ℃ for 5 min and then amplified over 45 cycles of 95 ℃ for 5s and 60 ℃ for 15s. Gene expression levels were quantified using the comparative Ct (ΔΔCt) method. The Ct values of target genes *NtIvnVR1_S/T* were normalized to the Ct value of the endogenous control gene *NtGS2*.

### Field design, RS, and total alkaloids analyses

All field evaluations were conducted at Clayton, North Carolina (USA) using a randomized complete block design with individual plants as the experimental units (39–50 plants per genotype). Agronomic practices typical for Virginia tobacco production, including topping, were employed. The plants were grown for 145 days from transplant to harvest time, after which they were cured for 8 days. Cured leaf tissues were analyzed for total alkaloid (TA) and RS content at the North Carolina State University Tobacco Analytical Service Laboratory, as previously described^94^.

### Starch measurement

Starch content was measured using the STARCH UV method described in Boehringer Mannheim/R-Biopharm; Enzymatic BioAnalysis/Food Analysis Cat. No. 10 207 748 035 https://food.r-biopharm.com/wp-content/uploads/2012/06/roche_ifu_starch_en_10207748035_2017-07.pdf. For samples that contained starch content above 30 g/100 g, a polarimetric internal method was used. The results were expressed as a percentage of total DW.

### Carbohydrate and chlorophyll measurements of greenhouse-grown plants

The sugars Fru, Glc, and Suc were measured using the method described in^95^. Briefly, the sample was extracted with water and diluted, followed by anion exchange chromatography analysis with a pulsed amperometric detector (HPAEC-PAD), and using an internal reference standard. The limit of quantification was 0.1 g/100 g for all analytes. The results were expressed as a percentage of total DW. The holistic method was used to estimate the measurement uncertainty of the analysis. The measurement of uncertainty for the sum of the sugars was obtained based on the uncertainties of the individual sugars. Chlorophyll was determined on fresh leaves using SPAD (the data correspond to the mean of 3 mid-stalk leaf measurements, *p* < 0.01, Student’s t-test).

### Free aa measurement

The total aa measured were: γ-aminobutyric acid, α-aminobutyric acid, aspartic acid, glutamic acid, alanine, arginine, asparagine, cysteine, cystine, citrulline, phenylalanine, phosphoserine, glycine, glutamine, hydroxylysine, hydroxyproline, isoleucine, histidine, leucine, lysine, methionine, ornithine, proline, serine, taurine, tyrosine, threonine, valine, and tryptophan After the samples were extracted, an internal standard was added, and the final solution was analyzed using ion exchange or reversed phase chromatography by post-column ninhydrin derivatization^96,97^. The results were expressed in mg/kg DW.

### Statistical analysis

Statistical analyses were performed using R software (version 4.2.3). For sugars, chlorophyll, starch content, and targeted gene expression analyses, comparisons between groups were performed using Student’s t-test, as these datasets followed approximately normal distributions. For transcriptomic analyses of the invertase gene family during the curing time course, gene expression levels were quantified as FPKM (Fragments Per Kilobase of transcript per Million mapped reads), and statistical significance was assessed using q-values derived from false discovery rate (FDR) correction to account for multiple testing. For metabolomics datasets, where sample sizes were limited (*n* = 3 or *n* = 8 per group) and data did not meet parametric assumptions, non‑parametric Kruskal–Wallis tests were applied, followed by appropriate post hoc comparisons. Unless otherwise stated, statistical significance was defined as *P* < 0.05 or q < 0.05. The specific statistical test used for each experiment is indicated in the corresponding figure legends.

## Supplementary Information

Below is the link to the electronic supplementary material.

Supplementary Material 1

Supplementary Material 2

## Data Availability

The RNA‑seq datasets generated and analysed during the current study are publicly available in the European Nucleotide Archive (ENA) under accession numbers PRJEB75461 (sample S300286) and PRJEB75459 (sample S990019), accessible at: https://www.ebi.ac.uk/ena/browser/view/PRJEB75461https://www.ebi.ac.uk/ena/browser/view/PRJEB75459.
