# Supplementary material for: Involvement of NtInvVR1 in the increase of reducing sugars during the curing of virginia tobacco
Source: Sci Rep. 2026 Apr 22;16:18755. doi: 10.1038/s41598-026-49747-x (PMC13273079; doi:10.1038/s41598-026-49747-x)
Supplement: Supplementary file 2 — Supplementary Material 2 [file 41598_2026_49747_MOESM2_ESM.pptx]

## Slide 1
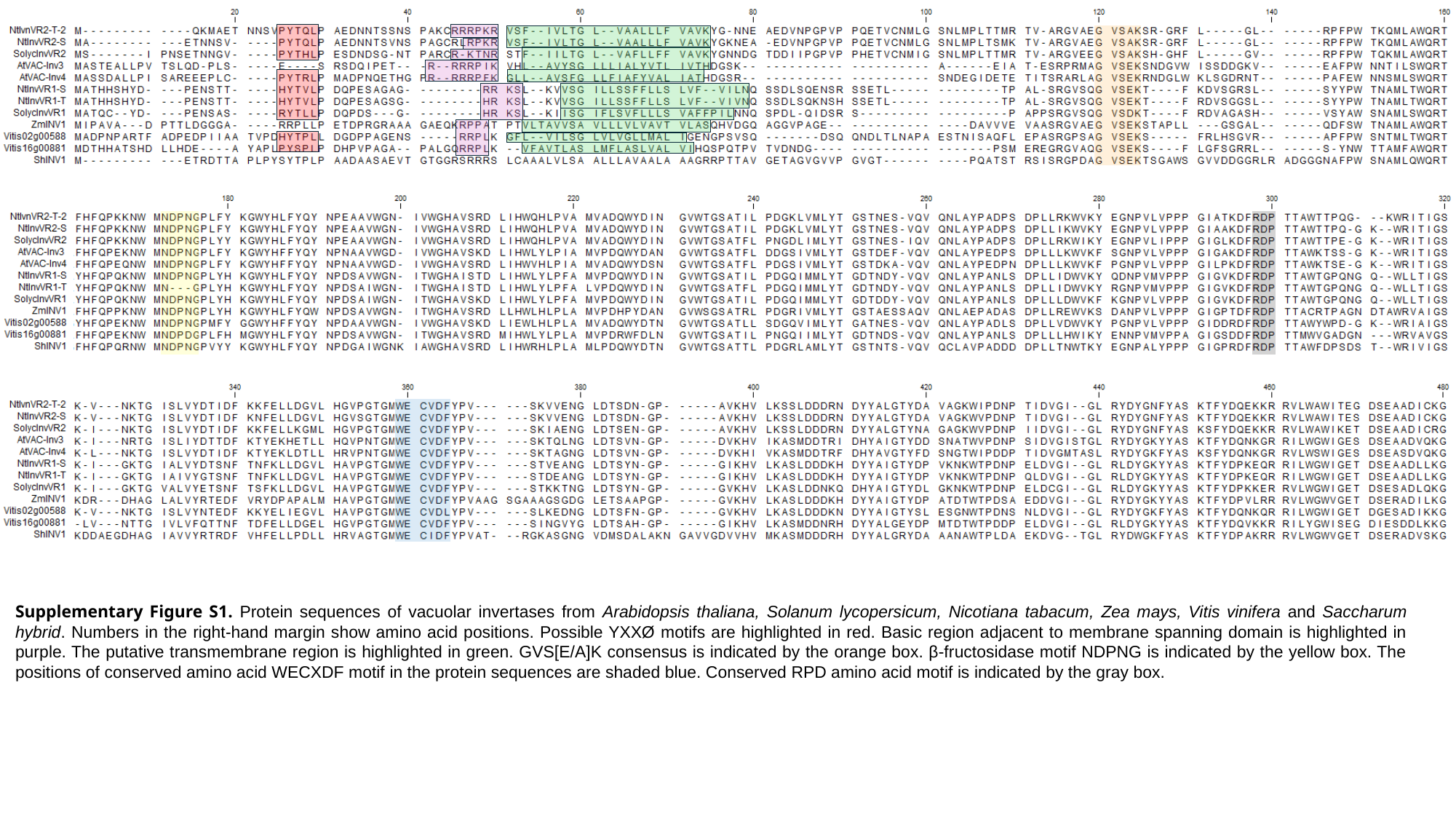

Supplementary Figure S1. Protein sequences of vacuolar invertases from Arabidopsis thaliana, Solanum lycopersicum, Nicotiana tabacum, Zea mays, Vitis vinifera and Saccharum hybrid. Numbers in the right-hand margin show amino acid positions. Possible YXXØ motifs are highlighted in red. Basic region adjacent to membrane spanning domain is highlighted in purple. The putative transmembrane region is highlighted in green. GVS[E/A]K consensus is indicated by the orange box. β-fructosidase motif NDPNG is indicated by the yellow box. The positions of conserved amino acid WECXDF motif in the protein sequences are shaded blue. Conserved RPD amino acid motif is indicated by the gray box.

## Slide 2
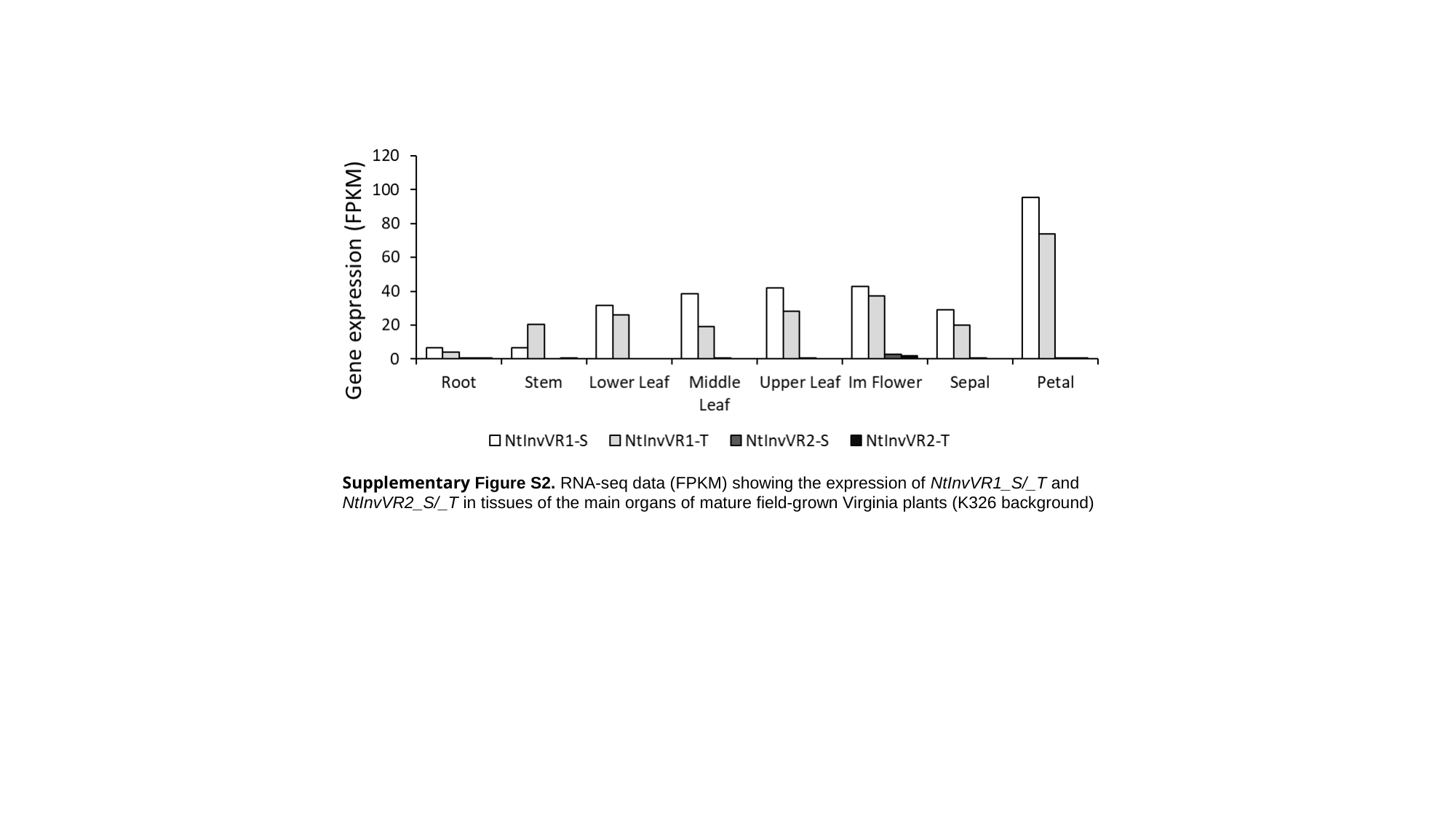

Supplementary Figure S2. RNA-seq data (FPKM) showing the expression of NtInvVR1_S/_T and NtInvVR2_S/_T in tissues of the main organs of mature field-grown Virginia plants (K326 background)

## Slide 3
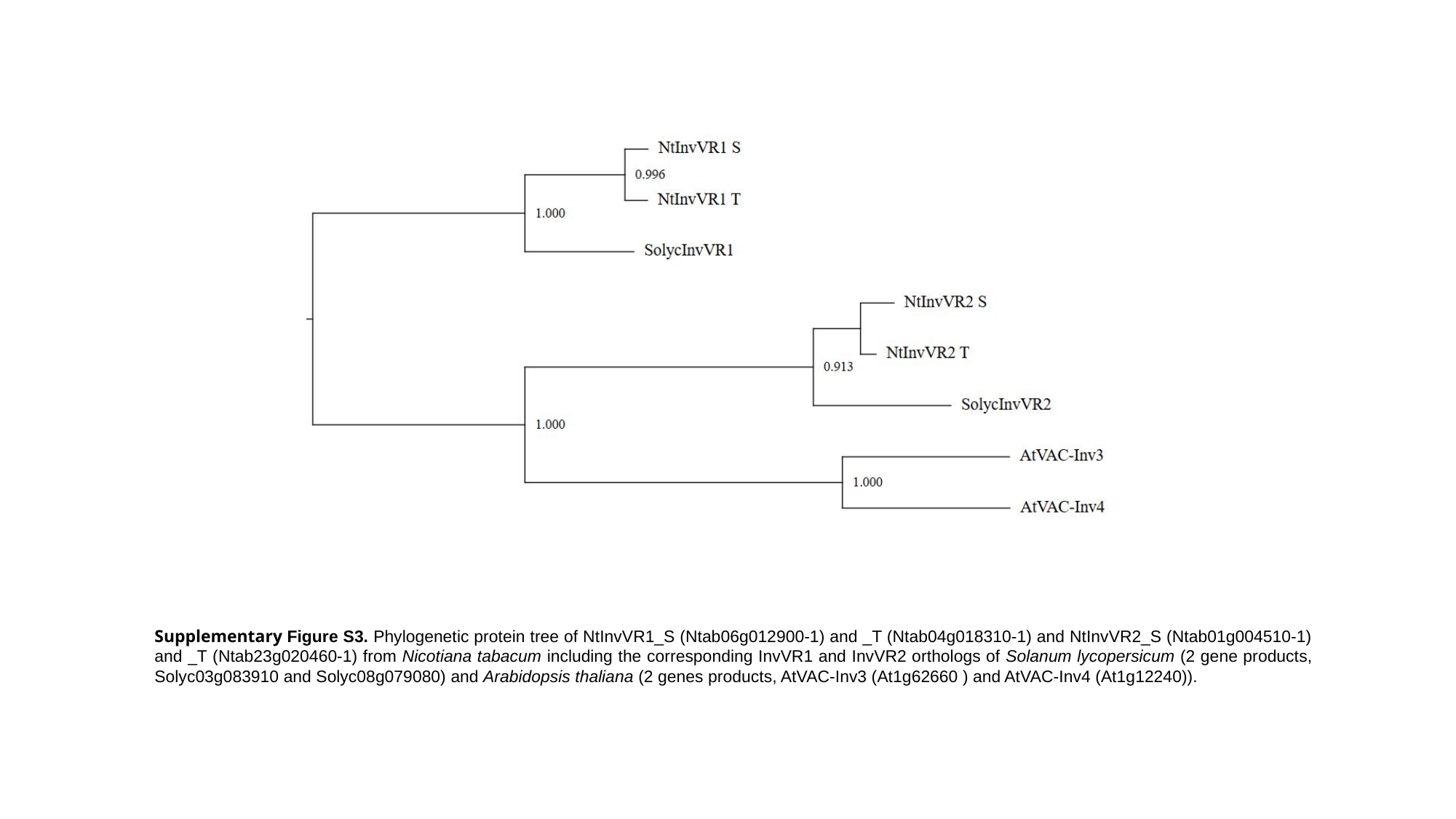

Supplementary Figure S3. Phylogenetic protein tree of NtInvVR1_S (Ntab06g012900-1) and _T (Ntab04g018310-1) and NtInvVR2_S (Ntab01g004510-1) and _T (Ntab23g020460-1) from Nicotiana tabacum including the corresponding InvVR1 and InvVR2 orthologs of Solanum lycopersicum (2 gene products, Solyc03g083910 and Solyc08g079080) and Arabidopsis thaliana (2 genes products, AtVAC-Inv3 (At1g62660 ) and AtVAC-Inv4 (At1g12240)).
